# Investigation of Dynamic Viscoelastic Characteristics of Permeable Asphalt

**DOI:** 10.3390/ma17122984

**Published:** 2024-06-18

**Authors:** Xin Yan, Zhigang Zhou, Zhiren Liu, Yang Zhou

**Affiliations:** 1 School of Traffic & Transportation Engineering, Changsha University of Science and Technology, Changsha 410114, China; yanxin@stu.csust.edu.cn (X.Y.); liuzhiren2024@163.com (Z.L.); zhouy@stu.csust.edu.cn (Y.Z.); 2 National Key Laboratory of Green and Long-Life Road Engineering in Extreme Environment, Changsha 410114, China

**Keywords:** permeable asphalt mixture, confining pressure, dynamic modulus, phase angle, main curve, Hirsch model

## Abstract

In order to provide a basis for the structural analysis, design and maintenance of permeable asphalt pavements, and to promote their engineering promotion and application, this study investigated the dynamic viscoelastic properties of permeable asphalt mixtures (PAC-13) under complex stress states. A Simple Performance Tester (SPT) system was used to measure the dynamic modulus of the mix under complex stress states. The displacement factor and principal dynamic modulus curves were formed by fitting Sigmoidal functions and using 1stOpt (first optimization) software, the phase angle principal curves were further determined, and the dynamic modulus was predicted for the ambient phase (15–25 °C) using the Hirsch model. The results showed that the dynamic modulus of the mixtures decreases with an increasing temperature, and the maximum decrease in the dynamic modulus is 93% when the confining pressure is 100 kPa and the loading frequency is 10 Hz. The dynamic modulus increases with an increasing confining pressure and loading frequency, the maximum increase with an increasing confining pressure is 26.1% when the temperature is 25 °C and the loading frequency is 10 Hz, and the maximum increase with an increasing loading frequency is 411% when the temperature is 25 °C and the confining pressure is 100 Hz. The dynamic modulus has a strong frequency dependence at low temperatures, while it is stress-dependent at high temperatures. Meanwhile, based on the Hirsch model, a new modified prediction model was developed, which can well predict the dynamic modulus of permeable asphalt mixtures at room temperature.

## 1. Introduction

At present, most of China’s urban roads are made of densely graded mixture, which has a negative impact on the ecological environment. Due to its low permeability and low noise reduction capabilities, the urban heat island effect and noise pollution are becoming increasingly serious [1,2,3]. In order to achieve the sustainable development of urban construction, the construction of permeable asphalt pavement is considered a new of “sponge city” trend. Permeable asphalt mixture has the outstanding advantages of high anti-skid performance, low noise and good drainage performance, and it is a leader in the existing asphalt pavement technology [4,5]. The increase of the void ratio of permeable asphalt mixture increases its permeability but weakens its mechanical properties, so the permeable asphalt mixture must have excellent permeability and mechanical properties [6]. The dynamic modulus is the main parameter when analyzing the mechanical characteristics and structural design of asphalt pavements [7]. Currently, the dynamic modulus denoted in the Mechanical-Empirical Pavement Design Guidelines (MEPDG) is measured by uniaxial compressive dynamic modulus testing [8]. The asphalt pavement structure is often in a state of multiaxial stress in the field. Obviously, the triaxial dynamic modulus of compression provides a more objective evaluation of asphalt pavement’s mechanical properties compared to the uniaxial dynamic modulus. Numerous studies have been conducted on asphalt mixture dynamic properties by scholars worldwide.

In China, in the study of the uniaxial compressive dynamic modulus of dense-graded asphalt mixtures, Lei Yu et al. [9] have conducted uniaxial dynamic modulus experiments to evaluate the dynamic modulus and the phase angle of rubberized asphalt mixture by various oil-to-rock ratios. The experiments were conducted at four temperatures and nine loading frequencies. They successfully established the dynamic modulus master curve for the rubberized asphalt mixture. Their findings indicate that the rubberized asphalt mixture’s dynamic modulus decreases with a rising temperature and diminishes with a decreasing loading frequency. The rubberized asphalt mixture exhibits the highest dynamic modulus and superior high-temperature stability at the optimal oil-to-rock ratio. The dynamic modulus master curve, with a 20 °C reference temperature, shows a good fit to the test data. Yanqing Zhao et al. [1] measured the dynamic moduli of SMA-13 and Superpave-20 at various loading frequencies and temperatures using an SPT. They analyzed the loading and temperature effects on the phase angle and dynamic modulus of these two types of asphalt mixture. Additionally, they developed master curves for the phase angle and dynamic modulus based on experimental data. The Witczak model was employed to perform predictive regression analysis of the master curves, and the predictions were found to be better. Qian Zhang et al. [10] measured the phase angle and dynamic modulus of three asphalt mixture: stone mastic asphalt (SMA-13), Superpave polyurethane mix (SUPU-20) and stone matrix polyurethane (SMPU-13). These measurements were carried out using the AMPT test. The findings indicate viscoelastic behavior with predominantly elastic properties in the two dense polyurethane mixes, and they demonstrate greater mechanical stability compared to traditional asphalt mixes under normal pavement operating temperatures and loading frequencies. Several factors affect the dynamic modulus, such as the test temperature, mixture type and confining pressure.

In foreign countries, there was a study on the uniaxial compressive dynamic modulus of dense-graded asphalt mixtures with external additives, ASMA. Rahman et al. [11] examined the effect of various warm-mix asphalt (WMA) and hot-mix asphalt (HMA) technologies on the asphalt mixture dynamic modulus. They established master curves for the phase angle and dynamic modulus of the warm-mix asphalt mixture. The results demonstrate a significant effect of the WMA agent on the asphalt mixture phase angle δ and dynamic modulus |E|. A.K. Arshad et al. [12] used nanosilica-modified asphalt to enhance the permeable asphalt mixture dynamic modulus and thus improve their rutting resistance. Mohammad Javad Amirkhani et al. [13] explored the effects of calcium carbonate fillers, fly ash, and Portland cement type II on the performance of warm-mix asphalt incorporating the Kaowax additive. Dynamic modulus experiments were performed to explore the effects. The findings indicate that the inclusion of the Kaowax additive increased the asphalt mixture dynamic modulus.

Through extensive research conducted both domestically and internationally, it is evident that numerous scholars have focused on investigating the conventional dense-graded asphalt mixture dynamic modulus. However, few research reports are available on the permeable asphalt mixture dynamic modulus, which poses limitations for pavement structure designs. Permeable asphalt mixture, being a large-porosity pavement structure, differs from conventional dense-graded asphalt mixture, especially under triaxial conditions. It is still to be explored whether it is about the dynamic modulus test method and data analysis, the method of establishing the dynamic modulus master curve, or the dynamic modulus prediction model. Therefore, the objective of the current study is to systematically evaluate the variations in the permeable asphalt mixture dynamic modulus under the temperature effect, confining the pressure and loading frequency. Through comprehensive dynamic modulus testing, we intend to develop phase angle and dynamic modulus master curves. These findings will serve as a foundation for predicting the permeable asphalt mixture phase angle and dynamic modulus over a wider range of frequency conditions and temperatures. In addition, the applicability of the Hirsch prediction model to predict the dynamic modulus of permeable asphalt mixtures will also be verified. If it is not applicable, we will be able to build a new modified prediction model based on the Hirsch model for the dynamic modulus of permeable asphalt mixture. This endeavor will contribute valuable insights for permeable asphalt mixture design and construction, facilitating its practical application in various contexts.

The main innovations of this paper are as follows:

1. Based on the Hirsch model, a new modified prediction model was developed to predict the dynamic modulus of permeable asphalt mixtures.
2. Permeable asphalt mixtures at high temperatures have significant stress dependence, and the presence of a confining pressure leads to a reduction in the temperature sensitivity of permeable asphalt mixtures at high temperatures.
3. Considering the mechanical properties of permeable asphalt mixtures under complex stress conditions, it is more realistic to reflect the mechanical response of the pavement under actual stress conditions.

## 2. Raw Materials and Gradation Design

### 2.1. Raw Material

The test asphalt is made of East China Sea Petroleum SBS-modified asphalt, provided by Xinyue Foshan Asphalt Co. (Foshan, China) This kind of asphalt itself is not easy to soften at high temperatures, and at low temperatures the brittle phenomenon appears, with the main technical indicators provided in Table 1, such that all the performance indicators meet “The Technical Code for Highway Asphalt Pavement Construction (JTG F40-2017)” requirements. The high-viscosity additives are produced and developed by a French pavement material industrial company, with yellow spherical particles at room temperature, and Table 2 summarizes the specific parameters. Also, the ratio is 92% SBS-modified asphalt + 8% high-viscosity modifier recommended by the manufacturer, prepared using an in-house real hair process. The specific steps are as follows: high-viscosity modifier and stone latex mixer, dry mixing 90 s, then add SBS-modified asphalt mixing 90 s, and finally, add mineral powder mixing 90 s, heating, mixing temperature of 180 °C. The coarse and fine aggregates are made of pyroxene, which is an igneous rock, and this kind of rock has good mechanical properties, durability, chemical stability and resistance to abrasion. Limestone is used for the mineral powder. Generally speaking, limestone is a more ideal material for mineral powder, and the aggregates are provided by Changsha Junjia Materials Co. (Changsha, China). The technical requirements of the aggregates are shown in Table 3 and Table 4.

### 2.2. Grading Design

PAC-13, which is commonly used in permeable pavement, is adopted as the research object and the void ratio of porous asphalt mixture at home and abroad is generally 18%~25%. Too large a void ratio is conducive to drainage, but it will reduce the mechanical properties, and with too low a void ratio, the mechanical properties are improved, but it will affect the drainage. Therefore, to take into account the mechanical properties, durability and drainage performance, it was decided to use 20% as the void ratio of asphalt pavement [15]. From the existing research, it can be seen that the expected target porosity is mainly obtained by adjusting and controlling the 2.36 mm sieve perforation throughput, and the median value of the gradation and the ±3% on the basis of it are generally regarded as the primary gradation [16]. The initial asphalt dosage is obtained according to the asphalt membrane thickness calculation formula presented in the Technical Code for “Highway Asphalt Pavement Construction (JTG F40-2017)” [17], as shown in Equations (1) and (2).

(1) A=(2+0.02a+0.04b+0.08c+0.14d+0.3e+0.6f+1.6g)/48.74,

(2) $$P_{b}=h\times A,$$

where A is the aggregate total surface area; a, b, c, d, e, f, g are 4.75, 2.36, 1.18, 0.06, 0.3, 0.15, and 0.075 mm sieve through the percentage, %; h stands for the asphalt film thickness (h = 14 μm); and Pb stands for the initial asphalt dosage, %.

**Figure 1:**
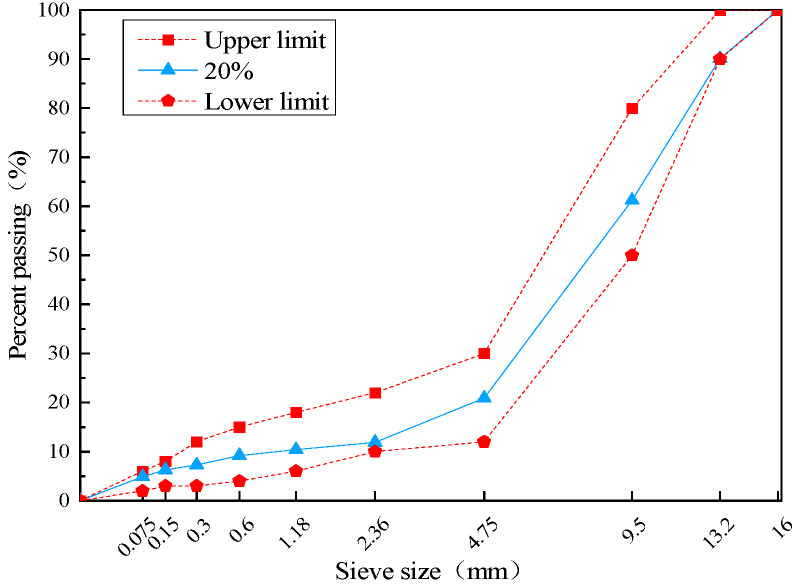
Grading curves of the base grades.

The specimen was formed by the rotational compaction method, the porosity was measured by the volumetric method and compared with the target porosity, and the target gradation was obtained after appropriate adjustment, as shown in Figure 1.

### 2.3. Optimal Asphalt Content

**Figure 2:**
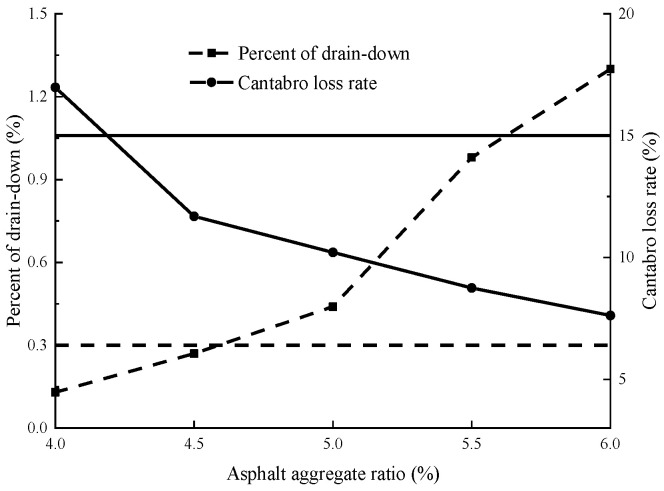
Optimal asphalt content determination.

Based on the “Highway Engineering Asphalt and Asphalt Mixture Test Procedures” (JTG E20-2011), the mixture was prepared according to five asphalt aggregate ratios of 4, 4.5, 5, 5.5, and 6%, and it was subjected to Schellenberg binder drainage and Cantabro tests, and the findings of the tests are illustrated in Figure 2.

The optimal asphalt binder content is obtained as shown in Equation (3).

OAC = OAC_min_ + 0.75 (OAC_max_ − OAC_min_), (3)

where OAC is the optimal content of asphalt binder, %; OAC_max_ is the maximum content of asphalt binder according to the Schellenberg binder drainage test, %, and OAC_min_ is the minimum content of asphalt binder according to the Cantabro test, %. 

To ensure compliance with the “Technical Specification for Permeable Asphalt Pavements” (CJJ T190-2012) [18] and “Technical Specification for Highway Asphalt Pavement Construction” (JTG F40-2017), it is critical to verify whether the optimal asphalt dosage and design gradation meet the specified requirements. Following the guidelines outlined in the “Standard Test Methods of Bitumen and Bituminous Mixture for Highway Engineering” (JTG E20-2011), a comprehensive set of tests was conducted: the Cantabro test, Schellenberg binder drainage test, rutting test, water immersion Marshall test, and freeze–thaw splitting test. Table 5 presents the test parameters and results, and all of them conform to the specified requirements.

## 3. Dynamic Modulus Test 

**Figure 3:**
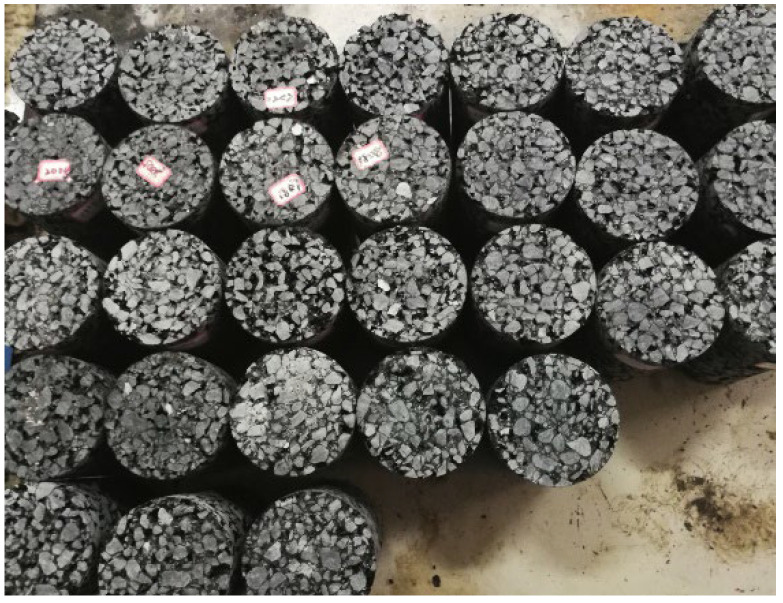
SPT test specimens.

Based on the specifications outlined in the “Standard Test Methods of Bitumen and Bituminous Mixture for Highway Engineering” (JTG E20-2011), the rotary compaction apparatus is utilized to fabricate cylindrical samples with a height and diameter of 170 and 150 mm, respectively. Subsequently, core samples of Φ100 mm × 150 mm dimensions are extracted from these specimens. The specimen target void ratio is set at 20 ± 0.5%, and a group consists of six parallel specimens. Some specimens are shown in Figure 3.

**Figure 4:**
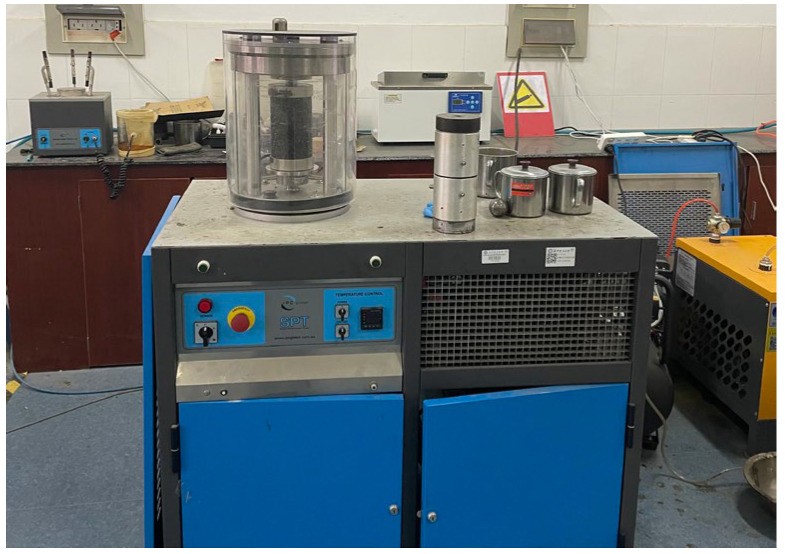
SPT test instruments.

According to the dynamic modulus test requirements in AASHTO TP62-2007 [19], the test temperature is 4, 15, 25, 40, and 55 °C. Combined with the actual force of the road surface, three confining pressures of 0, 100, and 200 kPa are selected, and the loading frequency is 0.1, 0.5, 1, 5, 10 and 20 Hz. As shown in Figure 4, the test instrument is selected from the SPT tester produced by the Australian IPC company. The instrument is capable of carrying out an asphalt mixture dynamic modulus test, repeated loading permanent deformation test, etc. The equipment consists of an electro-hydraulic loading system, a perimeter pressure system, an environmental chamber and a corresponding control system, using air as the perimeter pressure. Before the test, the specimen is put into the specified constant temperature box insulation for 4 h, turning on the instrument power system, and keeping the temperature of the test chamber to the specified temperature. The displacement sensor is installed in the middle of the side of the specimen according to the specification, put on the rubber membrane, and fixed in the test chamber. Under the specified temperature conditions, the specimen can be applied with controllable sinusoidal pressure of different frequencies, the load value is 13.5 kN, and the strain control mode is adopted with strain range of 85–115 με. Then, the dynamic modulus of resilience test begins. The test sequence is from low temperature to high temperature, the same temperature, and from high frequency to low frequency. The dynamic modulus and phase angle are expressed as Equations (4) and (5).

(4) $$\left|E^{*}\right|=\frac{\sigma_{o}}{\varepsilon_{o}},$$

(5) $$\varphi =\frac{T_{i}}{T_{p}}(360),$$

where ε_o_ is the strain curve amplitude; $\sigma_{o}$ is the stress curve amplitude; φ is the phase angle; $T_{p}$ is the action time of the applied stress; |E*| is the dynamic modulus; and $T_{i}$ is the time lag between stress and strain.

## 4. Analysis of Test Results

### 4.1. Effect of Temperature on Phase Angle and Dynamic Modulus

**Figure 5:**
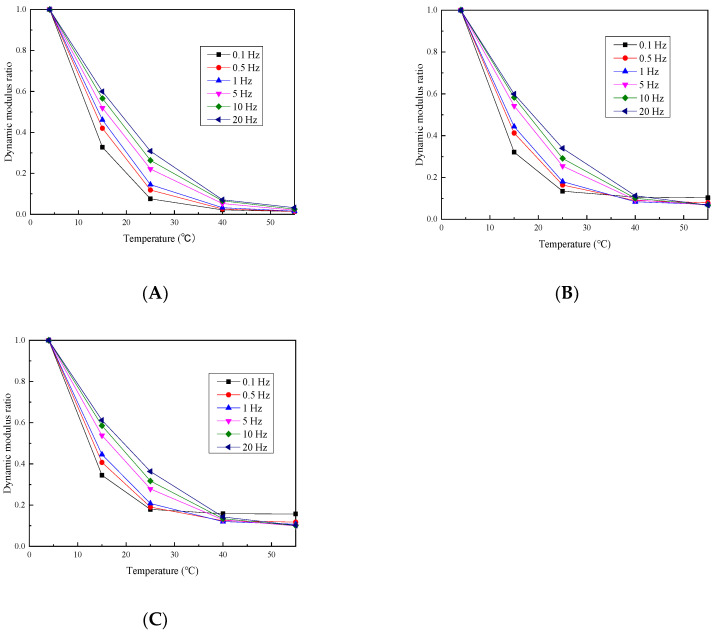
Dynamic modulus ratio under various confining pressure values of (**A**) 0, (**B**) 100 and (**C**) 200 kPa.

**Figure 6:**
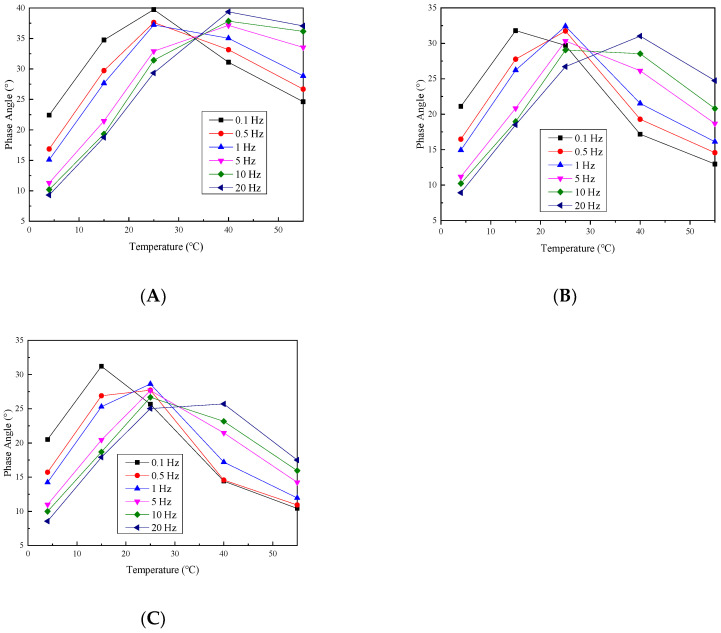
Phase angle under different confining pressure values of (**A**) 0, (**B**) 100 and (**C**) 200 kPa.

To provide a clearer understanding of the temperature–dynamic modulus relationship, the values of the dynamic modulus at 4 °C are used as a reference. The ratio of the dynamic modulus value at each temperature to the reference value is calculated under various confining pressures, and the corresponding trends are illustrated in Figure 5 and Figure 6.

Under the three confining pressures and six loading frequencies, the permeable asphalt mixture dynamic modulus ratio shows a consistent trend with the temperature, contracting as the temperature rises. Furthermore, the rate of shrinking decreases with higher confining pressures. This could be due to the increasing softening of the asphalt binder as the temperature rises, resulting in enhanced viscosity that hampers elastic recovery. Consequently, the overall elastic recovery capacity of the mixture diminishes, decreasing the dynamic modulus. As the confining pressure increases, however, the interlocking effect among the aggregate particles in the mixture intensifies. This effect slows down the degradation of the elastic recovery capacity, thereby causing the dynamic modulus to decrease at a slower rate. Under the three confining pressures and the six loading frequencies, the phase angle of the permeable asphalt mixture exhibits a pattern of first increasing and then reducing with the increase in temperature. This behavior can be because of the gradual enhancement of asphalt adhesion within the mixture as the temperature slowly increases. Therefore, the internal friction angle of the aggregate particles reduces, increasing the phase angle. However, with a further increase in the temperature, the asphalt adhesive properties diminish, and the mixture performance becomes primarily influenced by the aggregate skeletal structure. Consequently, the phase angle decreases. From the above analysis, it is clear that permeable asphalt mixtures have a significant stress dependence at high temperatures.

### 4.2. Influence of Confining Pressure on Phase Angle and Dynamic Modulus

**Figure 7:**
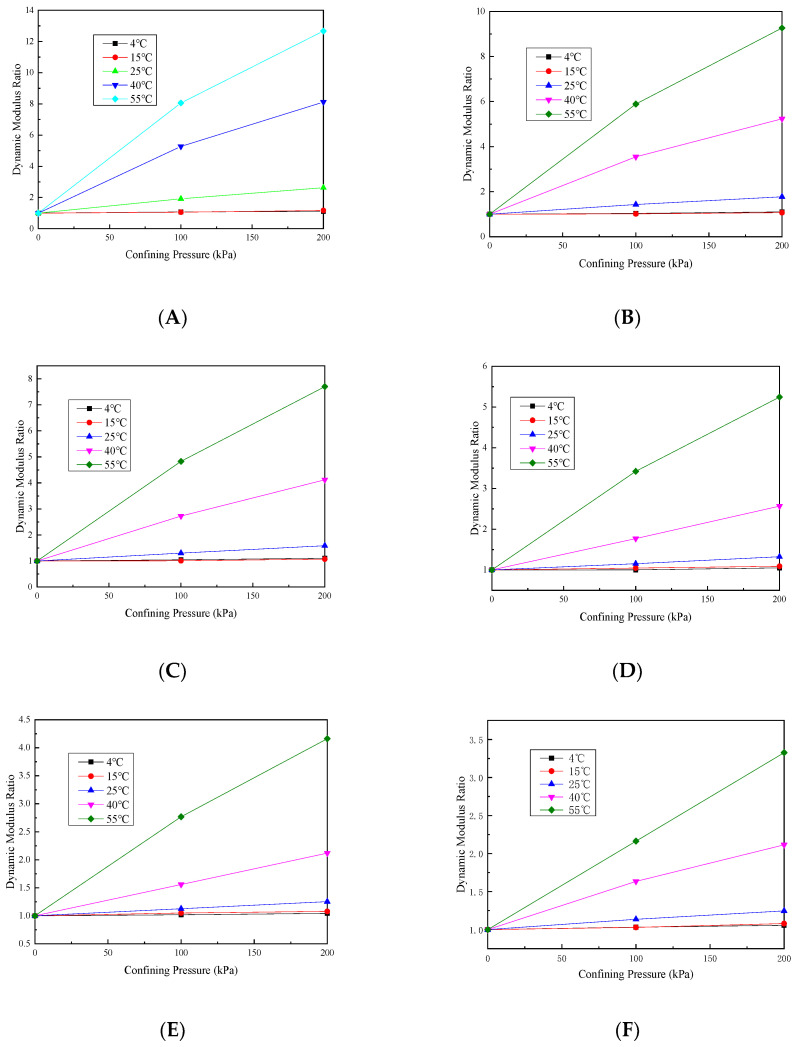
Dynamic modulus ratio under different loading frequency values of (**A**) 0.1, (**B**) 0.5, (**C**) 1, (**D**) 5, (**E**) 10 and (**F**) 20 Hz.

**Figure 8:**
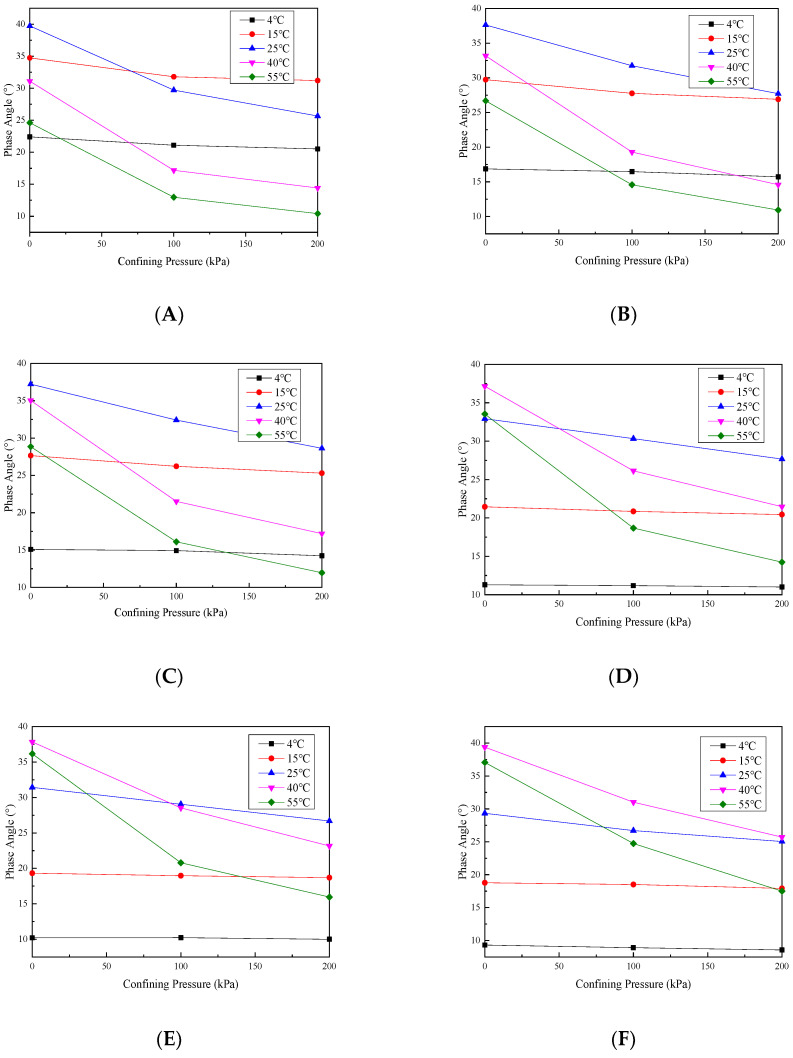
Phase angle under different loading frequency values of (**A**) 0.1, (**B**) 0.5, (**C**) 1, (**D**) 5, (**E**) 10 and (**F**) 20 Hz.

In order to see the change in the dynamic modulus with the confining pressure more intuitively, the dynamic modulus without a confining pressure is taken as the reference value, and the dynamic modulus/benchmark value ratio under different confining pressures is taken, and the change law is shown in Figure 7 and Figure 8.

Under the six loading frequencies, at temperatures below 15 °C, the dynamic modulus ratio exhibits a slight increase with an increasing confining pressure. However, the maximum ratio observed at a frequency as low as 0.1 Hz is only 1.11. This indicates that the confining pressure effect on the dynamic modulus is minimal and almost negligible when the temperature is below 15 °C. This is mainly because at temperatures below 15 °C, the asphalt adhesive properties within the mixture predominantly dictate its behavior, while the confining pressure slightly affects the overall mixture response.

As the temperature rises, the dynamic modulus ratio is increased in tandem by increasing the confining pressure, and the growth rate exhibits an exponential pattern with the increasing temperature. This can be due to the decreased adhesive properties of the asphalt within the mixture as the temperature rises. Consequently, the overall mixture performance becomes primarily influenced by the aggregate skeletal structure performance. Additionally, the increase in the confining pressure strengthens the interlocking effect among aggregates in the mixture. These observations indicate that under low-frequency and high-temperature conditions, the permeable asphalt mixture’s viscoelastic behavior is significantly influenced by the confining pressure. Therefore, in areas with high annual average temperatures, the impact of heavy load vehicles on permeable pavement must be considered when designing permeable pavement.

The change in the phase angle basically reduces as the confining pressure increases, and this decrease is small at low temperatures, but the reduction is very large at high temperatures. This is because as the confining pressure is increased, the aggregate intercalation effect in the mixture is gradually enhanced, so that the internal friction angle between the aggregates increases, so the increase in the confining pressure will decrease the phase angle. In addition, at low temperatures, the adhesion properties of asphalt change less, so the phase angle reduces less as the confining pressure increases. As the temperature is increased, the asphalt in the mixture gradually softens and the adhesion ability gradually weakens, and as the confining pressure is increased, the resistance of the mixture to deformation is gradually increased by the skeleton structure, and the phase angle reduction will also become larger.

### 4.3. Effect of Loading Frequency on Phase Angle and Dynamic Modulus

**Figure 9:**
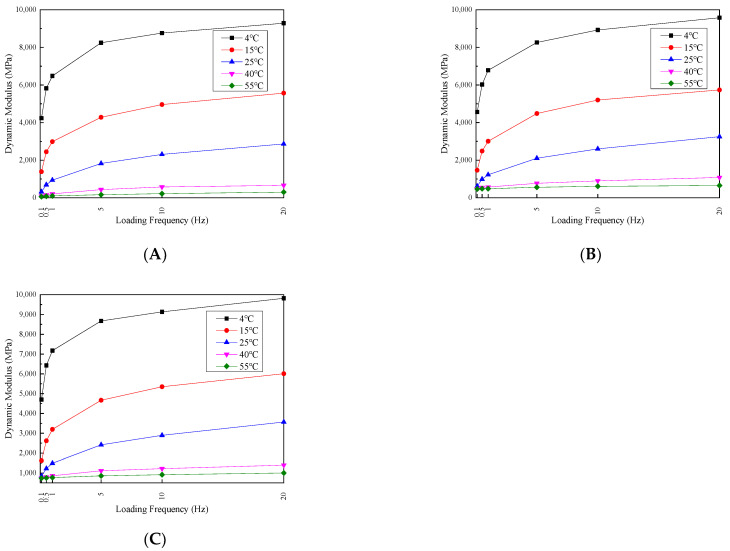
Dynamic modulus at confining pressure values of (**A**) 0, (**B**) 100 and (**C**) 200 kPa.

**Figure 10:**
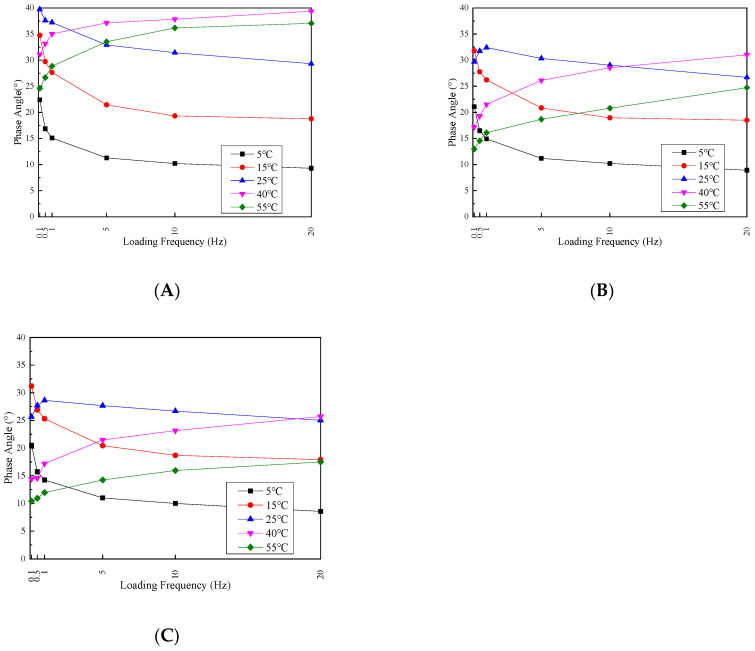
Phase angle at different confining pressure values of (**A**) 0, (**B**) 100 and (**C**) 200 kPa.

To analyze the loading frequency effects on the dynamic modulus and phase angle, the test result trends are depicted in Figure 9 and Figure 10.

The trend of the dynamic modulus in the permeable asphalt mixture exhibits a consistent pattern of increasing with the loading frequency across all three confining pressures and five temperatures. This is because of the enhanced elastic properties of the mixture by increasing the loading frequency, resulting in an overall increase in the dynamic modulus. However, the dynamic modulus–loading frequency relationship is temperature-dependent. In the 4 °C to 25 °C temperature range, the dynamic modulus experiences a sharp increase by increasing the loading frequency from 0.1 to 1 Hz, followed by a slower increase from 1 Hz to 20 Hz. Conversely, at temperatures ranging from 40 °C to 55 °C, the dynamic modulus demonstrates a relatively uniform rate of increase with the rise in the loading frequency. These observations can be explained by the differences in the elastic recovery and response to load changes at different temperatures. At lower temperatures, the mixture exhibits better elastic recovery and response to load variations. However, as the temperature increases, the asphalt softening within the mixture leads to slightly weaker elastic recovery and a diminished response to load changes.

The permeable asphalt mixture’s phase angle exhibits significant variation with the loading frequency. By changing the temperature from 4 to 15 °C, the phase angle decreases as the loading frequency increases for all three confining pressure conditions. At 25 °C, the phase angle behavior is influenced by the confining pressure magnitude. With zero confining pressure, increasing the loading frequency decreases the phase angle. However, at 100 and 200 kPa confining pressures, the phase angle is initially increased and then decreased by increasing the loading frequency.

By increasing the temperature from 40 °C to 55 °C, the phase angle is enhanced with the increase in the loading frequency under all three confining pressure conditions. This can be attributed to the following reasons. At lower temperatures, with the increase in the frequency, the aggregate internal friction angle within the mixture also increases, decreasing the phase angle. As the temperature rises, the asphalt viscosity within the mixture increases, decreasing the aggregate internal friction angle. Consequently, the phase angle increases. However, the presence of confining pressure mitigates the phase angle. The increased internal friction angle between the aggregates induced by the confining pressure reduces the phase angle magnitude.

## 5. Main Curves of Phase Angle and Dynamic Modulus of Permeable Asphalt Mixtures 

Based on the existing research of this group, planning a solution method using the initial values recommended in the NCHRP9-29 program is not reliable, and the reliability of the solution results is not high when the optimal solution is solved using the planning solution function of EXCEL 2016 [20]. To prevent error due to the initial value qualification of the fitting, 1stopt 5.0 (first optimization) software is used for the fitting, which does not require a given initial value. The principle of the new method is that the fitting equation for the master curve is obtained by substituting the reference temperature of 25 °C as the fitting parameter d, as follows:

(6) $$\log\left|E^{*}\right|=\delta +\frac{{\log E^{*}}_{max}}{{1+e}^{\beta +\gamma \left(\log t-d*\left(\frac{1}{T+273.15}-\frac{1}{298.15}\right)\right)}},$$

where t is the loading time, s; $\left|E^{*}\right|$ is the asphalt mixture dynamic modulus, psi; δ is the curvilinear function limiting minimum, representing the limiting minimum dynamic modulus logarithm, psi; $\log E_{max}^{*}$ is the ultimate maximum dynamic modulus logarithm, psi; β, γ are parameters describing the waveform of the sigmoidal function, respectively, and d = $\Delta E_{a}/2.303R$; T is the test temperature.

The method requires only the test results and test condition parameters at the time of dynamic modulus determination, i.e., the measured dynamic modulus value, the test temperature and the test loading frequency, as well as the limiting maximum modulus value logE*_max_ = 6.3767 obtained by using the Hirsch model calculations and the reference temperature of 25 °C used to obtain the master curve for the advection. Using the measured dynamic modulus obtained at each test temperature and frequency, a database was established, and then the values of the shape parameters of the curves (δ, β, γ, d) were obtained by global optimization using the McCourt optimization method in the 1stopt software. The measured dynamic modulus and loading time were then logarithmized to form the master curve and the displacement factor required for the formation of the phase angle master curve could be directly used as a result of the dynamic modulus master curve when it was established.

### 5.1. Determination and Analysis of Main Dynamic Modulus Curve 

**Figure 11:**
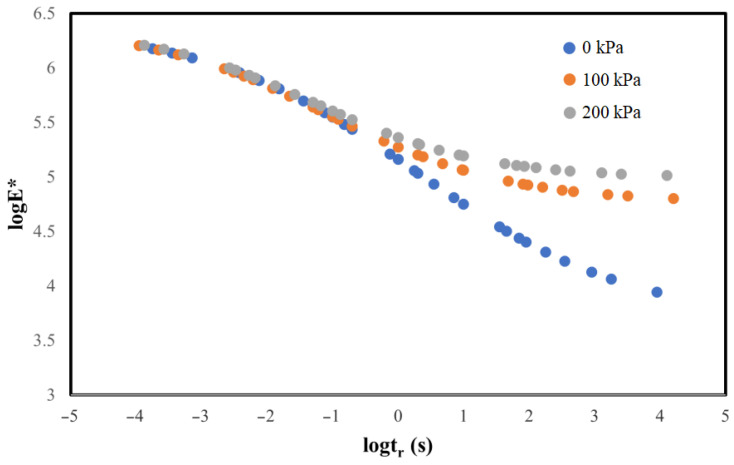
Comparison of the dynamic modulus main curves of the permeable asphalt mixture under three confining pressures.

**Figure 12:**
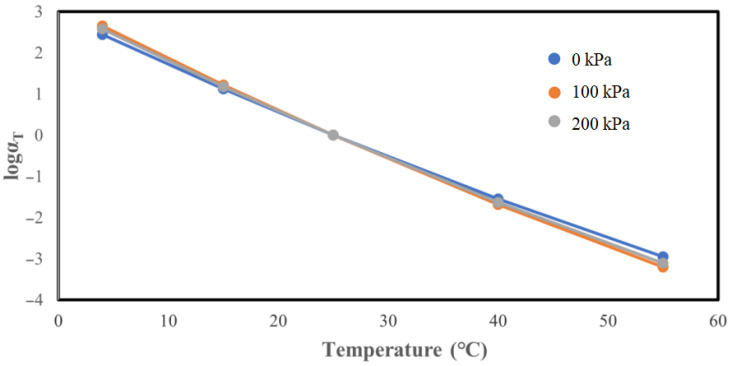
Comparison of the displacement factors under three confining pressures.

According to the findings of this research, the logarithms of the measured loading time and dynamic modulus are taken, and Equation (6) is used as the fitting function. The comparison of the shift factor and main curve under the three confining pressure conditions is shown in Figure 11 and Figure 12.

The goodness of fit for the dynamic modulus master curves of the permeable asphalt mixture was exceptionally high, exceeding 0.99, for all three confining pressure scenarios.

The limiting minimum modulus value in the primary curve of the permeable asphalt mixture’s fitted dynamic modulus exhibits a specific change gradient and is increased by increasing the confining pressure.

Across the three confining pressures, the displacement factor gradually decreases as the temperature increases. The displacement factor serves as a temperature sensitivity indicator for the mixture, suggesting that the permeable asphalt mixture’s temperature sensitivity intensifies with rising temperatures.

### 5.2. Determination and Analysis of Phase Angle Main Curve

**Figure 13:**
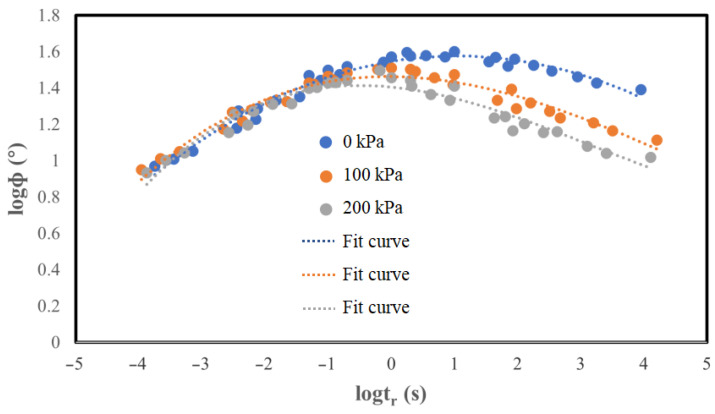
Comparison of the phase angle main curves under the three confining pressures.

According to the obtained experimental results, it was observed that the phase angle is initially increased and then decreased as the loading frequency changes. Consequently, phase angle master curve fitting using the Sigmoidal model, as performed for the dynamic modulus master curves, is not feasible. However, according to the viscoelastic theory of asphalt mixture, the shift factor represents the mixture’s temperature sensitivity and is solely related to the temperature. Consequently, the measured phase angle and dynamic modulus at the same temperature should correspond to the temperature sensitivity. Therefore, the displacement coefficient necessary for constructing the phase angle master curve is directly calculated from the master curve of the dynamic modulus. The corresponding data are illustrated in Figure 13.

The main phase angle curve after the shift factor translation can roughly represent the phase angle trend with the loading frequency and temperature, but the discrete type of phase angle is relatively large and the main curve is not smooth enough. The main curve of the phase angle without a confining pressure is smoother than that of the other two main curves with a confining pressure, and the distribution of points on the curve is more concentrated than that of the remaining two curves with a confining pressure. This illustrates that the confining pressure significantly affects the phase angle, that is, the confining pressure has a strong effect on the permeable asphalt mixture’s viscous properties.

The main curve of the phase angle exhibits a similar trend to that of the loading frequency and temperature across all three confining pressure cases, initially increasing and then decreasing. However, the position of the inflection point in the phase angle changes by increasing the confining pressure. Specifically, as the confining pressure increases, the phase angle inflection point initially rises and then falls. This reveals that the phase angle inflection point shifts toward higher loading frequencies with increasing confining pressure.

The phase angle main curve’s overall shape under the three confining pressures follows a clear hierarchy, and by increasing the confining pressure, the phase angle main curve tends to shift downwards. In other words, the magnitude of the phase angle is reduced by increasing the confining pressure. This can be attributed to the enhanced internal friction angle between the aggregates in permeable asphalt mixture resulting from the higher confining pressure, leading to a reduction in the phase angle.

## 6. Prediction Model of Permeable Asphalt Mixture Dynamic Modulus

At present, among the commonly used dynamic modulus estimation models, the Hirsch and Witczak models are more representative. The Hirsch model was originally developed by T.J. Hirsch based on an empirically determined constant approach for calculating the modulus of elasticity, aggregate modulus and cement MASTIC modulus, and mix properties of cement mixes and mortars. Later, Christensen et al. proposed an improved Hirsch model in 2003, which is a simplified predictive model where the model produces a simple expression based on the mixing rules, where the dynamic modulus of asphalt concrete is expressed in terms of the dynamic shear modulus of asphalt, the interstitial rate of the mineral aggregates, and the asphalt filler gap rate [21]. The Hirsch model has poor prediction accuracy at high temperatures and low-frequency phases but fair prediction accuracy at room temperature, and the Hirsch model requires fewer necessary inputs, is relatively simpler than the Witczak model, and is suitable for large-porosity asphalt mixtures. The Hirsch estimation model is used for validation and correction. Its mathematical form is as follows:

(7) $$|E^{*}|=P_{c}\left\{4200000\left(1-\frac{VMA}{100}\right)+3|G^{*}|\times \left(\frac{VFA\times VMA}{10000}\right)\right\}+\;\left(1-P_{c}\right)/\left[\frac{1-\frac{VMA}{100}}{4200000}+\frac{VMA}{3|G^{*}|\times (VFA)}\right]$$

(8) $$P_{c}=\frac{{\left[20+\frac{3|G^{*}|(VFA)}{VMA}\right]}^{0.58}}{650+[\frac{3|G^{*}|(VFA)}{VMA}{]}^{0.58}}$$

where $\left|G^{*}\right|$ is the asphalt binder dynamic shear complex modulus; $\left|E^{*}\right|$ is the mix dynamic modulus; VFA is the asphalt filler ratio; and VMA is the mineral aggregate void fraction.

**Figure 14:**
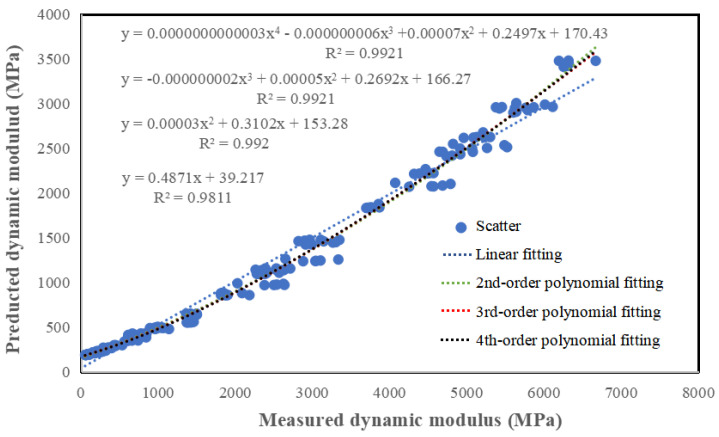
Dynamic modulus measured values fitted to the predicted values predicted dynamic modulus.

The required shear modulus in the Hirsch prediction model can be obtained by a dynamic shear rheometer, and the required VMA and VFA can be calculated from the gradation table and the oil/stone ratio. The final predicted and measured moduli are illustrated in Figure 14.

Figure 14 shows that the relationship between the predicted and measured values is very highly correlated using both linear fitting and polynomial fitting, with an R^2^ greater than 0.98, which is very reliable. By comparing the coefficient change of the highest order of the polynomial, it can be found that the coefficient value becomes very small with the increase in the order, so the relationship between the predicted and measured values can be expressed more accurately by using linear fitting. Therefore, the Hirsch model better predicts the permeable asphalt mixture dynamic modulus, but the predicted value needs to be corrected, and the expression of the correction is: y = 0.4871x + 39.217.

Since our specification takes the dynamic modulus at 10 Hz and 20 °C as a design parameter in designing the pavement structure, the Hirsch model can predict the permeable asphalt mixture dynamic modulus.

## 7. Conclusions

(1) The permeable asphalt mixture dynamic modulus is increased by increasing the confining pressure, and the growth rate becomes more pronounced as the temperature rises. On the other hand, the phase angle is decreased by increasing the confining pressure. At lower temperatures, the decrease is relatively small, but it becomes more significant at higher temperatures.
(2) The dynamic modulus of permeable asphalt mixtures reflects the significant thermo-rheological properties, frequency characteristics and stress dependence. The permeable asphalt mixture dynamic modulus is decreased by increasing the temperature, while the decrease rate diminishes with an increasing confining pressure. The phase angle exhibits an initial increase followed by a decrease with increasing temperature, and the confining pressure and loading frequency affect the peak value.
(3) The permeable asphalt mixture dynamic modulus is increased with an increasing loading frequency, with rapid increases observed at lower temperatures, which slow down as the temperature rises. The phase angle variation with the frequency is affected by both the temperature and confining pressure. Specifically, under the temperature range of 4–15 °C, the phase angle is decreased with an increasing loading frequency. At 25 °C, the phase angle change is associated with the magnitude of the confining pressure, decreasing with an increasing frequency in the absence of a confining pressure and exhibiting an initial increase followed by a decrease by increasing the loading frequency. By increasing the temperature from 40 °C to 55 °C, the phase angle is increased by increasing the loading frequency. As the dynamic modulus change of permeable asphalt mixtures under the complex stress situation is considered, it better reflects the mechanical properties of the pavement under the actual stress situation and provides a reference for the structural design of permeable asphalt mixtures. Thus, the reduction because of the structural design and the actual stress situation of the pavement is not the same as that brought about by the reduction of the life of the pavement.
(4) The Hirsch model is employed to predict the permeable asphalt mixture dynamic modulus, and it demonstrates a strong correlation with the measured values. In pavement structure design, when test data are unavailable, the Hirsch model can be utilized for the prediction of the dynamic modulus. It is recommended to use the modified predicted value as a representation of the final dynamic modulus value, with the modified expression given as: y = 0.4871x + 39.2173.
(5) Regarding the dynamic modulus prediction model for pervious asphalt mixtures, the Hirsch model cannot accurately predict it at high temperatures, and a suitable dynamic modulus prediction model for pervious asphalt mixtures needs to be found. The dynamic modulus and phase angle under wet conditions are of great importance for the validity of the presented results in this research. Next, we will investigate the dynamic modulus under wet conditions.

## Figures and Tables

**Table 1:** Basic performance indexes of SBS-modified asphalt.

Test Item	Unit	Technical Requirements	Test Methods	Test Results
Penetration grade 25 °C	0.1 mm	≥40	T0604	42.1
Softening point R&B	°C	≥80	T0606	86.3
Ductility 5 °C	cm	≥30	T0605	21.73

**Table 2:** Performance indexes of high-viscosity additives.

Test Item	Unit	Technical Requirements	Test Methods	Test Results
Appearance	-	Granular, even and full	JT/T 860.2 [14]	Qualified
Particle mass	G	≤0.03	JT/T 860.2	Qualified
Relative density	-	≤1.00	JT/T 860.2	Qualified
Melt index	g/10 min	≥2.0	JT/T 860.2	Qualified
Ash	%	≤2	JT/T 860.2	Qualified

**Table 3:** Technical specifications of coarse aggregates.

Test Item	Unit	Technical Requirements	Test Methods	Test Results
Stone crushing value	%	≤26	T0316	10.4
Los Angeles abrasion loss	%	≤28	T0317	11.6
Apparent relative density	-	≥2.6	T0304	2.83
Water absorption	%	≤2	T0304	1.82
Firmness	%	≤8	T0314	0.25
Acicular and flaky particle content	%	≤10	T0312	7.31
Washing method less than 0.075 mm particle content	%	≤1	T0310	0.3
Soft stone content	%	≤3	T0320	0.6

**Table 4:** Technical specifications of fine aggregates.

Test Item	Unit	Technical Requirements	Test Methods	Test Results
Apparent relative	-	≥2.5	T0328	2.76
Density	%	≥10	T0340	-
Firmness	%	≤1	T0333	0.2
Mud content	%	≥60	T0334	71
Sand equivalent	s	≥30	T0345	48

**Table 5:** Technical specifications of permeable asphalt mixtures.

Gap Ratio (%)	Optimal Asphalt Content (%)	Percent of Drain-Down (%)	Cantabro Loss Rate (%)	Dynamic Stability (Times)	Residual Stability (%)	Splitting Strength Ratio (%)
20	4.5	0.24	14	9265	88.3	84.4

## Data Availability

The data presented in this study are available on request from the corresponding author (due to privacy).
